# Supplementary material for: Epigenomic Features and Potential Functions of K+ and Na+ Favorable DNA G-Quadruplexes in Rice
Source: Int J Mol Sci. 2022 Jul 29;23(15):8404. doi: 10.3390/ijms23158404 (PMC9368837; doi:10.3390/ijms23158404)
Supplement: Supplementary file 1 [file ijms-23-08404-s001.zip › Supplementary data-2022-7-8-checked.pptx]

## Slide 1
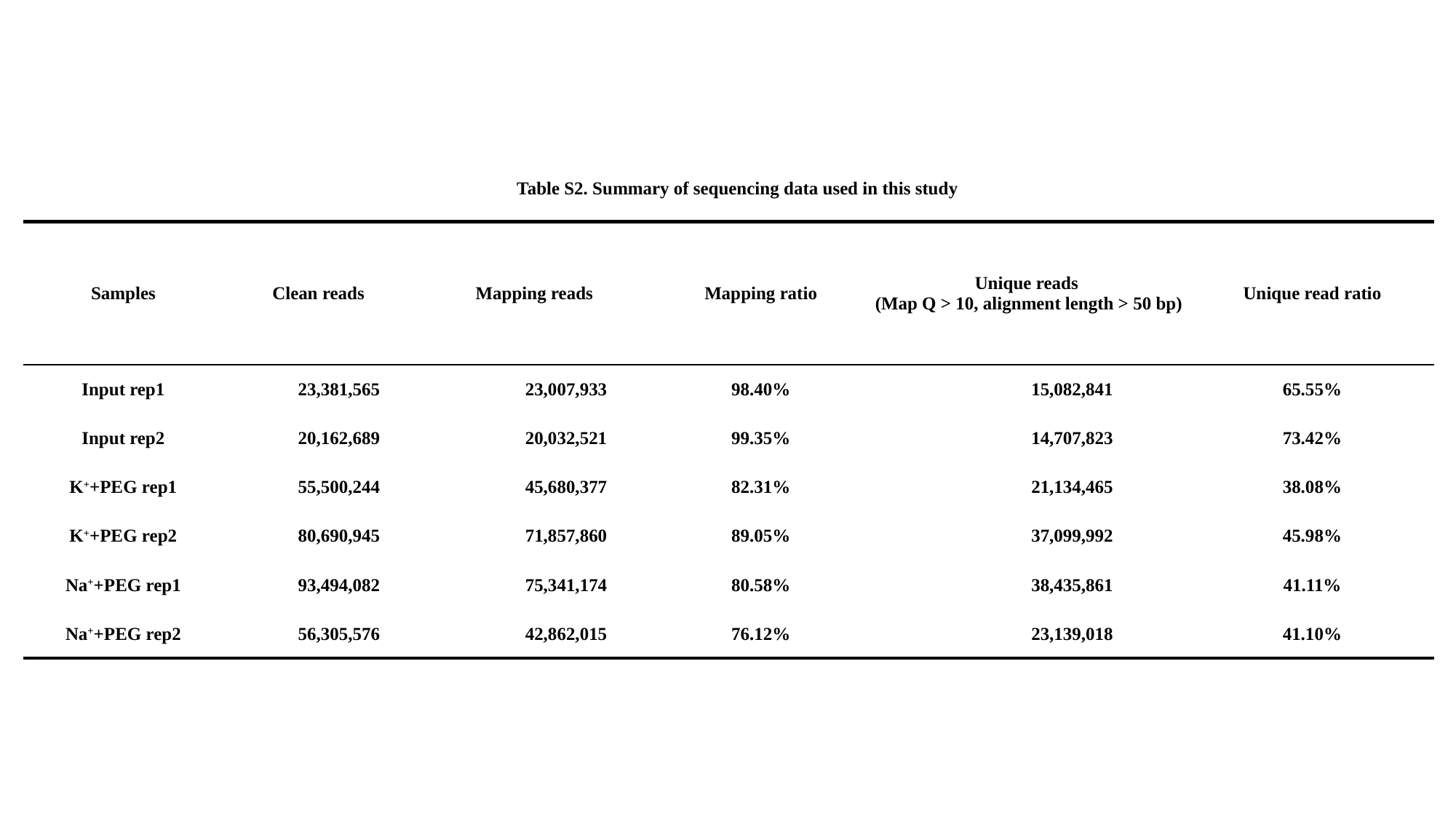

Table S2. Summary of sequencing data used in this study
| Samples | Clean reads | Mapping reads | Mapping ratio | Unique reads (Map Q > 10, alignment length > 50 bp) | Unique read ratio |
| --- | --- | --- | --- | --- | --- |
| Input rep1 | 23,381,565 | 23,007,933 | 98.40% | 15,082,841 | 65.55% |
| Input rep2 | 20,162,689 | 20,032,521 | 99.35% | 14,707,823 | 73.42% |
| K++PEG rep1 | 55,500,244 | 45,680,377 | 82.31% | 21,134,465 | 38.08% |
| K++PEG rep2 | 80,690,945 | 71,857,860 | 89.05% | 37,099,992 | 45.98% |
| Na++PEG rep1 | 93,494,082 | 75,341,174 | 80.58% | 38,435,861 | 41.11% |
| Na++PEG rep2 | 56,305,576 | 42,862,015 | 76.12% | 23,139,018 | 41.10% |

## Slide 2
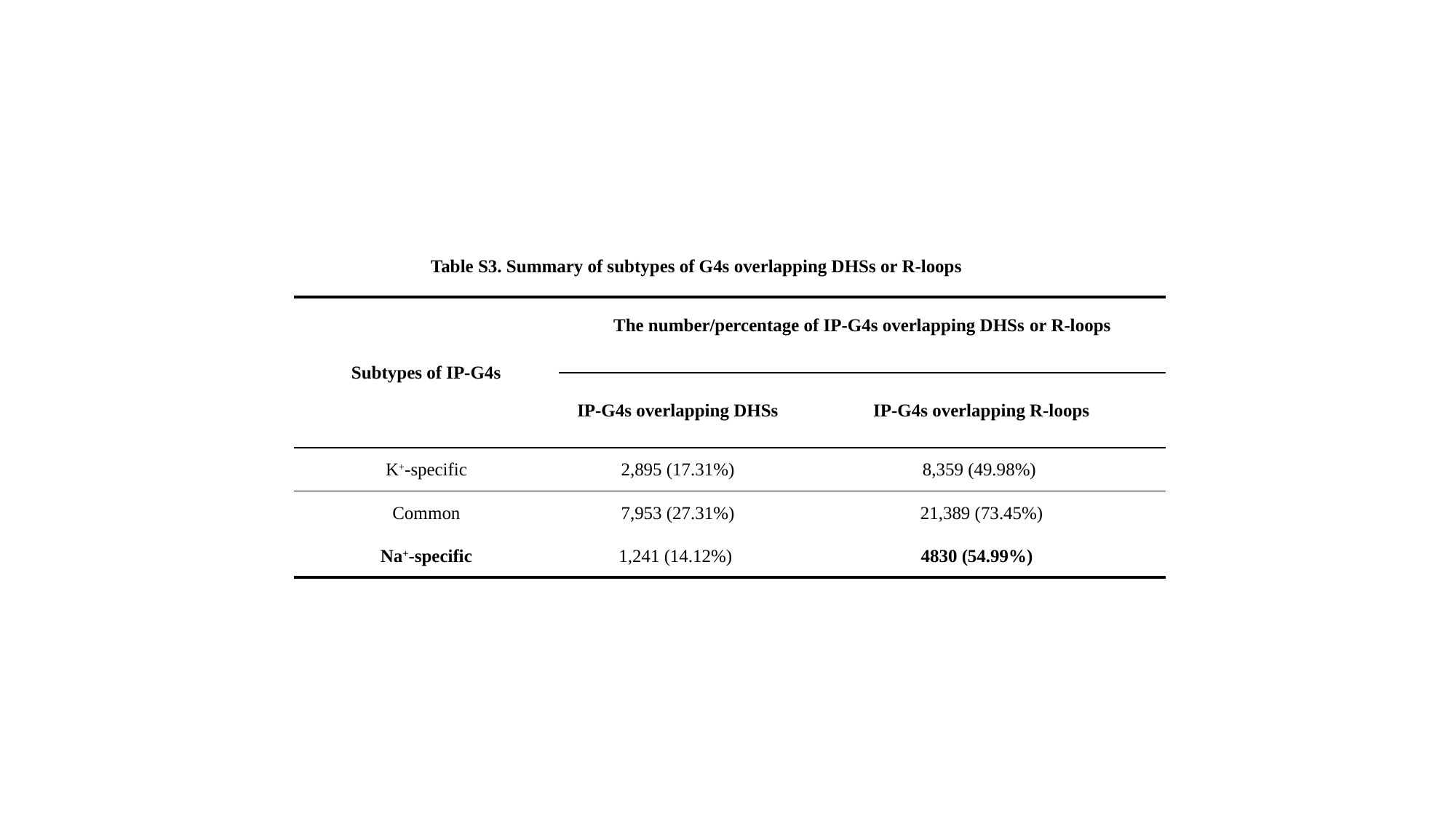

Table S3. Summary of subtypes of G4s overlapping DHSs or R-loops
| Subtypes of IP-G4s | The number/percentage of IP-G4s overlapping DHSs or R-loops | |
| --- | --- | --- |
| | IP-G4s overlapping DHSs | IP-G4s overlapping R-loops |
| K+-specific | 2,895 (17.31%) | 8,359 (49.98%) |
| Common | 7,953 (27.31%) | 21,389 (73.45%) |
| Na+-specific | 1,241 (14.12%) | 4830 (54.99%) |

## Slide 3
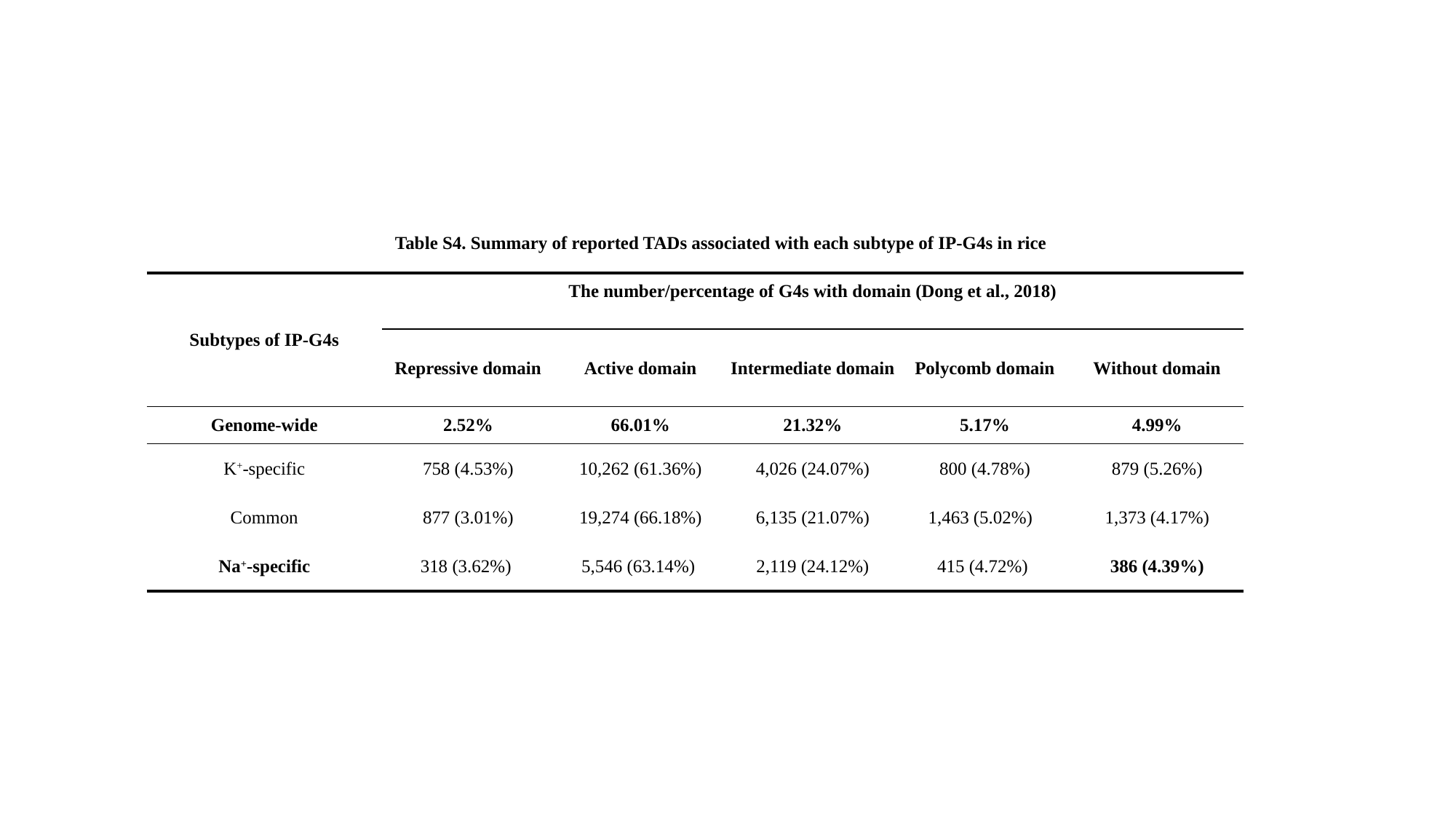

Table S4. Summary of reported TADs associated with each subtype of IP-G4s in rice
| Subtypes of IP-G4s | The number/percentage of G4s with domain (Dong et al., 2018) | | | | |
| --- | --- | --- | --- | --- | --- |
| | Repressive domain | Active domain | Intermediate domain | Polycomb domain | Without domain |
| Genome-wide | 2.52% | 66.01% | 21.32% | 5.17% | 4.99% |
| K+-specific | 758 (4.53%) | 10,262 (61.36%) | 4,026 (24.07%) | 800 (4.78%) | 879 (5.26%) |
| Common | 877 (3.01%) | 19,274 (66.18%) | 6,135 (21.07%) | 1,463 (5.02%) | 1,373 (4.17%) |
| Na+-specific | 318 (3.62%) | 5,546 (63.14%) | 2,119 (24.12%) | 415 (4.72%) | 386 (4.39%) |

## Slide 4
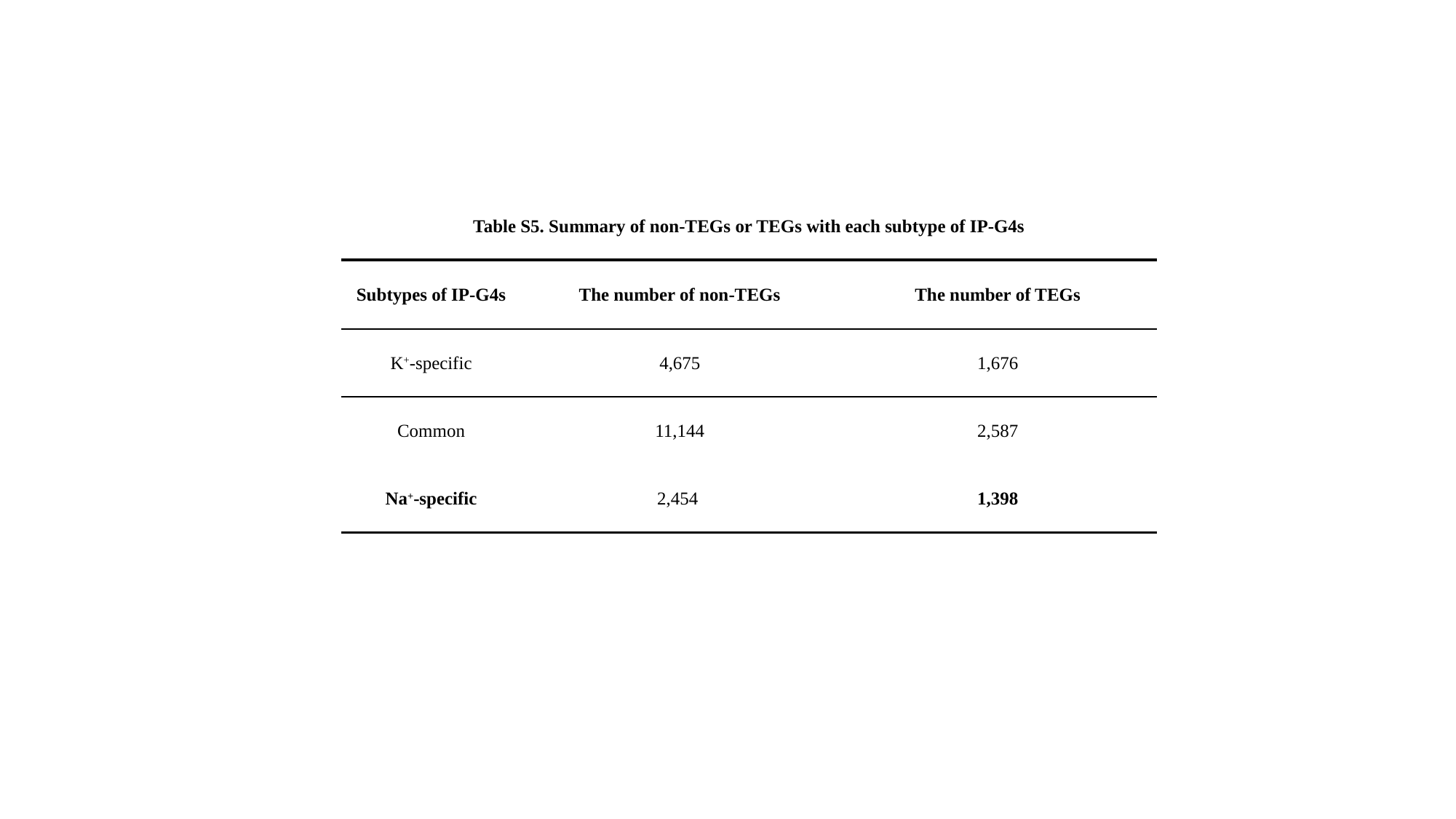

Table S5. Summary of non-TEGs or TEGs with each subtype of IP-G4s
| Subtypes of IP-G4s | The number of non-TEGs | The number of TEGs |
| --- | --- | --- |
| K+-specific | 4,675 | 1,676 |
| Common | 11,144 | 2,587 |
| Na+-specific | 2,454 | 1,398 |

## Slide 5
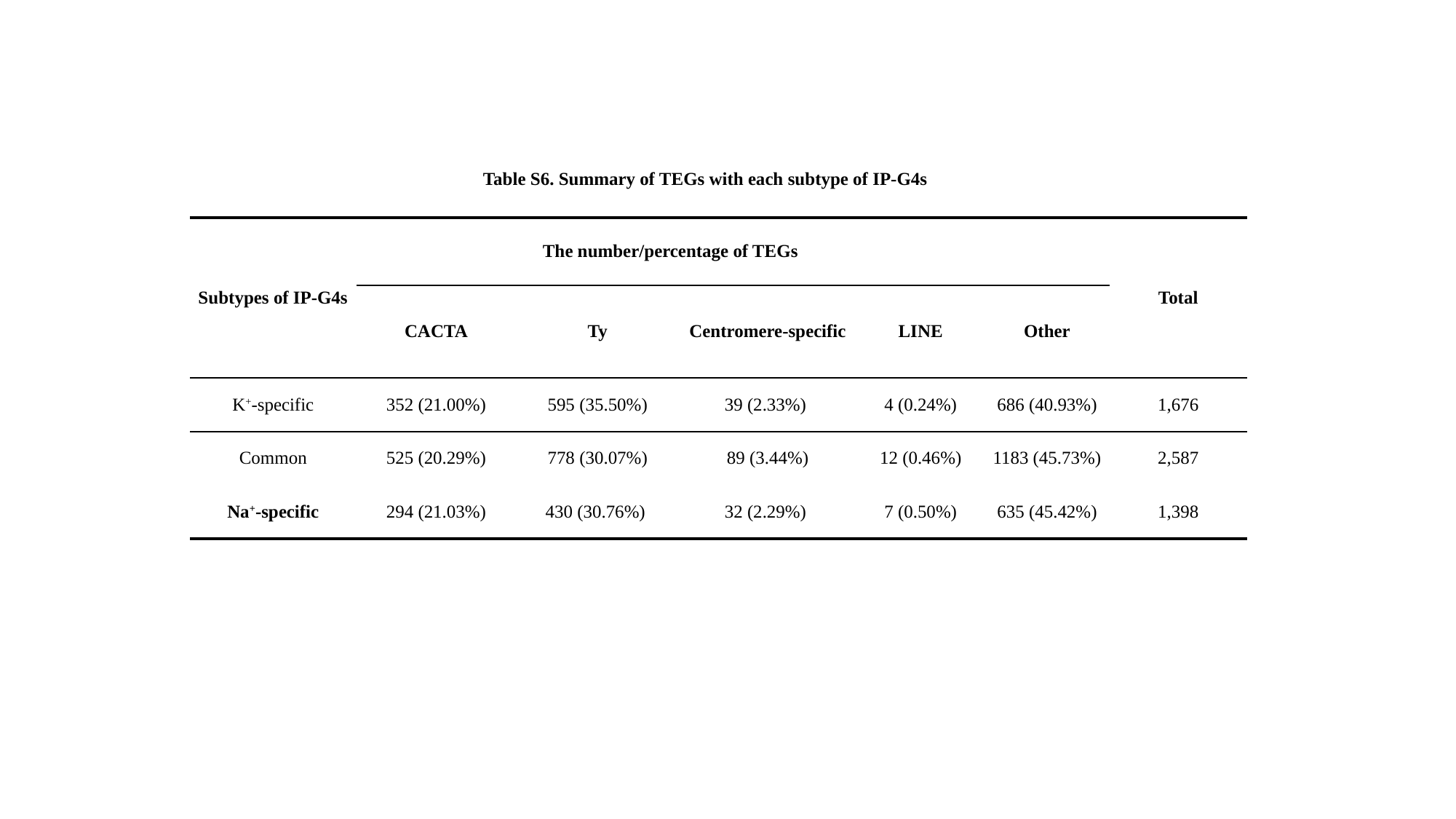

Table S6. Summary of TEGs with each subtype of IP-G4s
| Subtypes of IP-G4s | The number/percentage of TEGs | | | | | Total |
| --- | --- | --- | --- | --- | --- | --- |
| | CACTA | Ty | Centromere-specific | LINE | Other | |
| K+-specific | 352 (21.00%) | 595 (35.50%) | 39 (2.33%) | 4 (0.24%) | 686 (40.93%) | 1,676 |
| Common | 525 (20.29%) | 778 (30.07%) | 89 (3.44%) | 12 (0.46%) | 1183 (45.73%) | 2,587 |
| Na+-specific | 294 (21.03%) | 430 (30.76%) | 32 (2.29%) | 7 (0.50%) | 635 (45.42%) | 1,398 |

## Slide 6
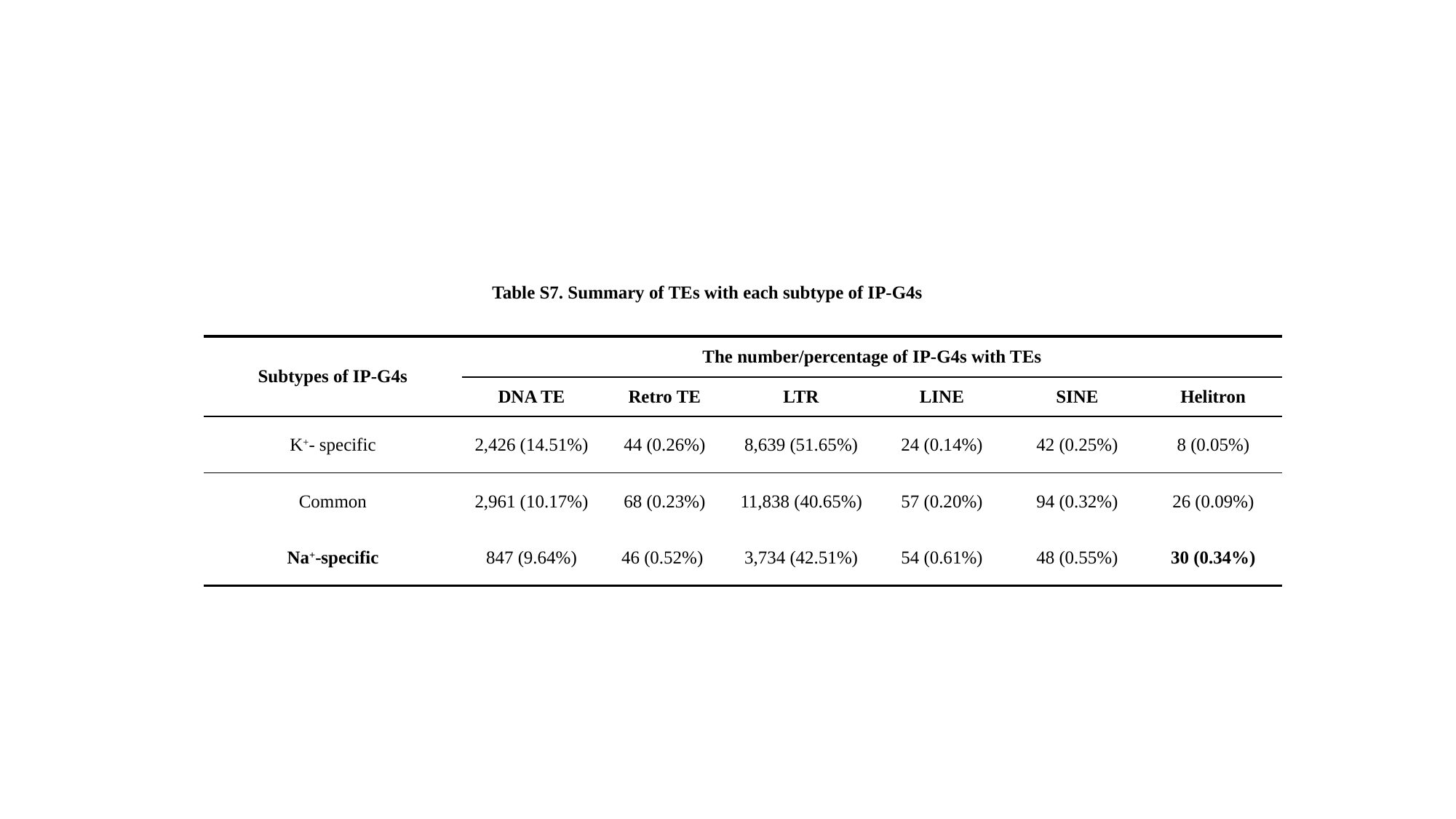

Table S7. Summary of TEs with each subtype of IP-G4s
| Subtypes of IP-G4s | The number/percentage of IP-G4s with TEs | | | | | |
| --- | --- | --- | --- | --- | --- | --- |
| | DNA TE | Retro TE | LTR | LINE | SINE | Helitron |
| K+- specific | 2,426 (14.51%) | 44 (0.26%) | 8,639 (51.65%) | 24 (0.14%) | 42 (0.25%) | 8 (0.05%) |
| Common | 2,961 (10.17%) | 68 (0.23%) | 11,838 (40.65%) | 57 (0.20%) | 94 (0.32%) | 26 (0.09%) |
| Na+-specific | 847 (9.64%) | 46 (0.52%) | 3,734 (42.51%) | 54 (0.61%) | 48 (0.55%) | 30 (0.34%) |

## Slide 7
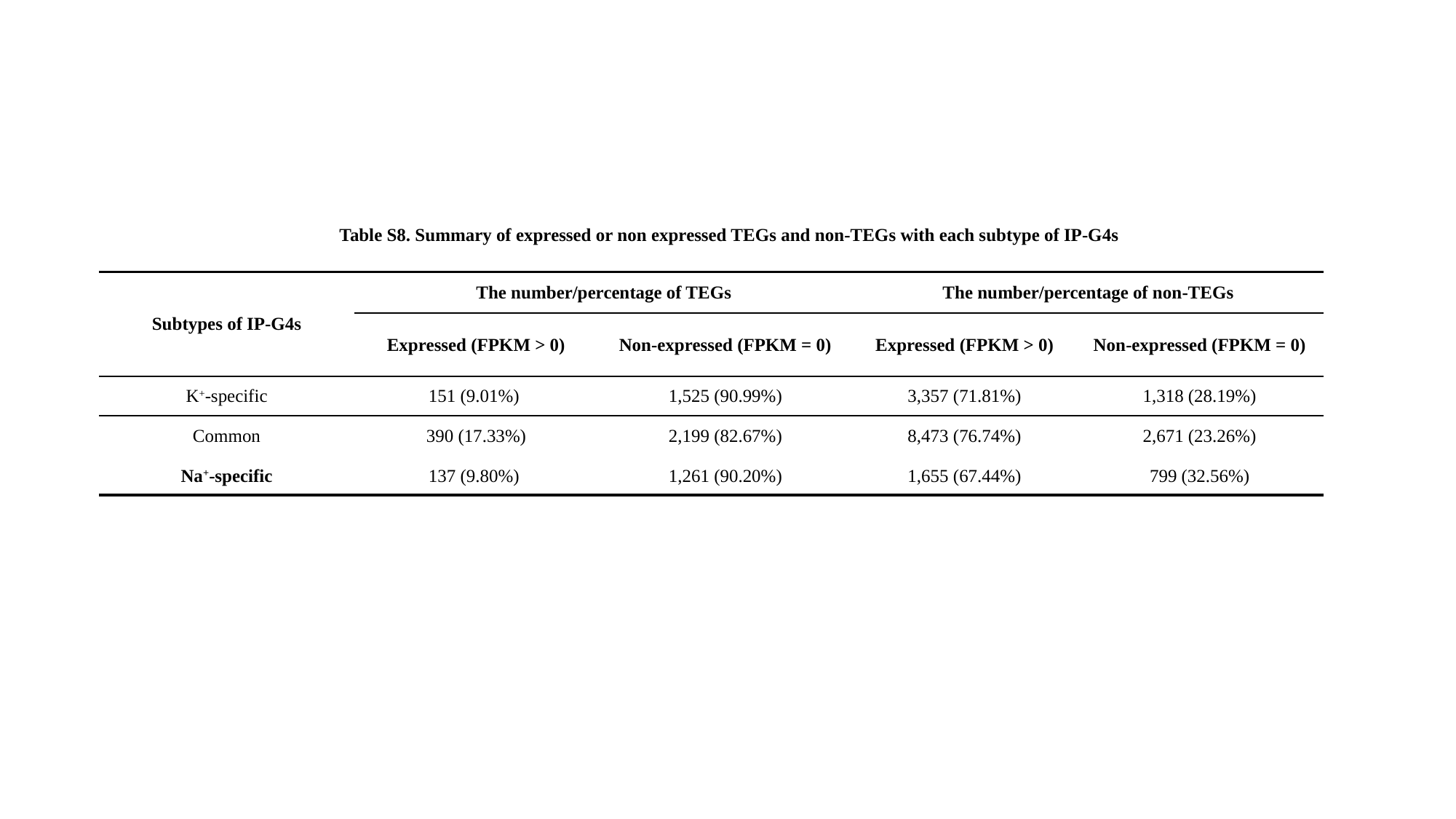

Table S8. Summary of expressed or non expressed TEGs and non-TEGs with each subtype of IP-G4s
| Subtypes of IP-G4s | The number/percentage of TEGs | | The number/percentage of non-TEGs | |
| --- | --- | --- | --- | --- |
| | Expressed (FPKM > 0) | Non-expressed (FPKM = 0) | Expressed (FPKM > 0) | Non-expressed (FPKM = 0) |
| K+-specific | 151 (9.01%) | 1,525 (90.99%) | 3,357 (71.81%) | 1,318 (28.19%) |
| Common | 390 (17.33%) | 2,199 (82.67%) | 8,473 (76.74%) | 2,671 (23.26%) |
| Na+-specific | 137 (9.80%) | 1,261 (90.20%) | 1,655 (67.44%) | 799 (32.56%) |

## Slide 8
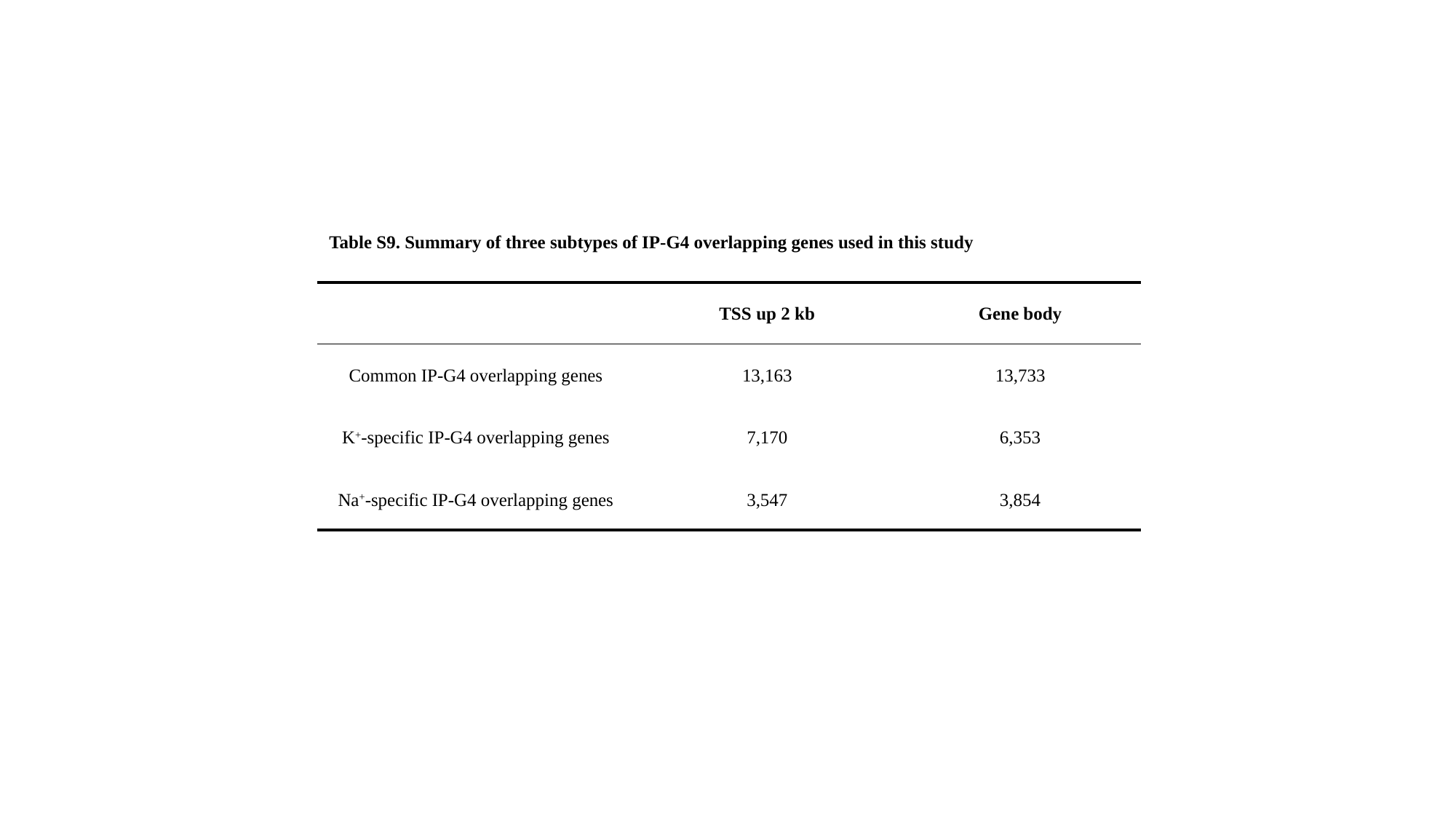

Table S9. Summary of three subtypes of IP-G4 overlapping genes used in this study
| | TSS up 2 kb | Gene body |
| --- | --- | --- |
| Common IP-G4 overlapping genes | 13,163 | 13,733 |
| K+-specific IP-G4 overlapping genes | 7,170 | 6,353 |
| Na+-specific IP-G4 overlapping genes | 3,547 | 3,854 |

## Slide 9
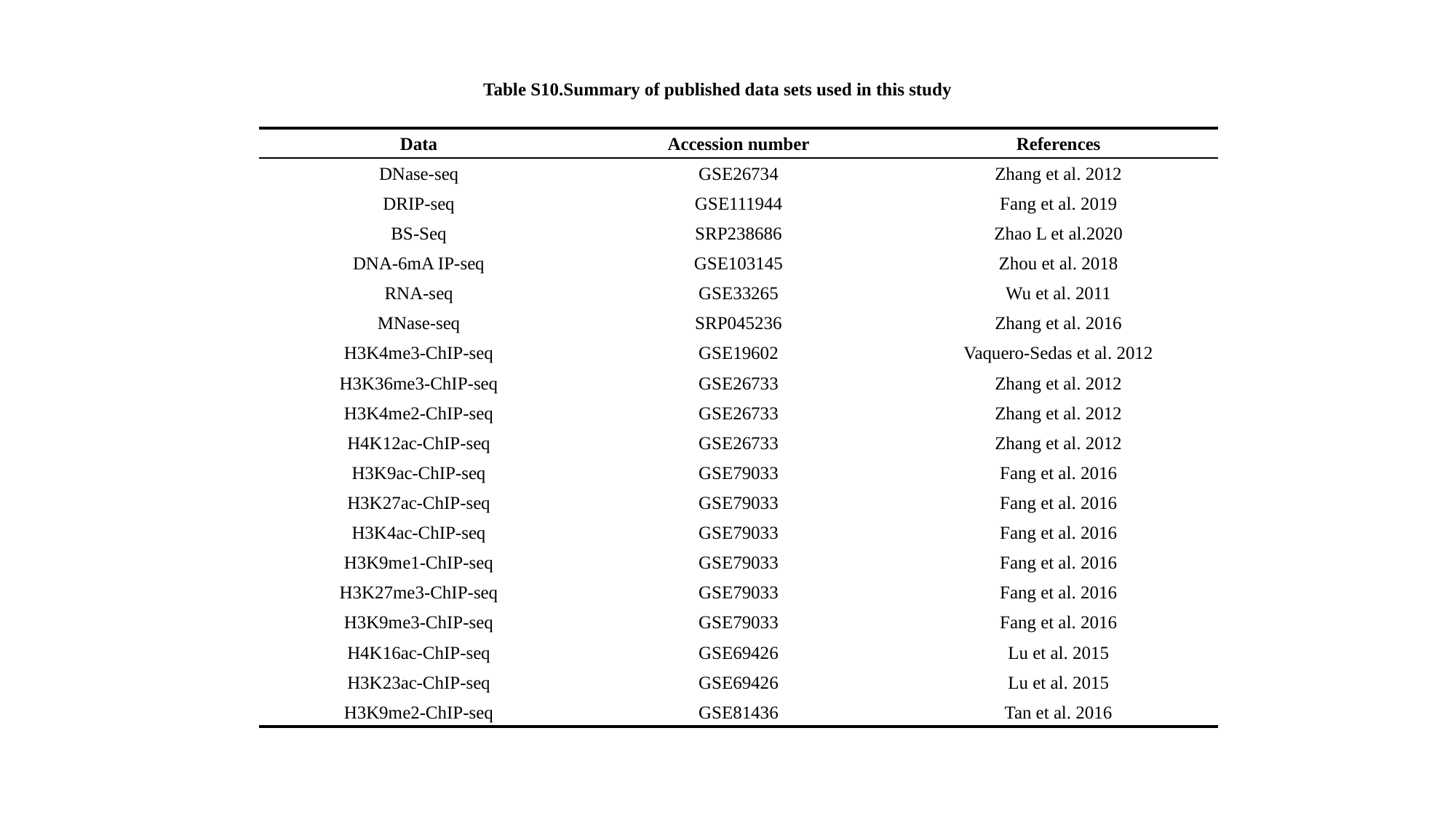

Table S10.Summary of published data sets used in this study
| Data | Accession number | References |
| --- | --- | --- |
| DNase-seq | GSE26734 | Zhang et al. 2012 |
| DRIP-seq | GSE111944 | Fang et al. 2019 |
| BS-Seq | SRP238686 | Zhao L et al.2020 |
| DNA-6mA IP-seq | GSE103145 | Zhou et al. 2018 |
| RNA-seq | GSE33265 | Wu et al. 2011 |
| MNase-seq | SRP045236 | Zhang et al. 2016 |
| H3K4me3-ChIP-seq | GSE19602 | Vaquero-Sedas et al. 2012 |
| H3K36me3-ChIP-seq | GSE26733 | Zhang et al. 2012 |
| H3K4me2-ChIP-seq | GSE26733 | Zhang et al. 2012 |
| H4K12ac-ChIP-seq | GSE26733 | Zhang et al. 2012 |
| H3K9ac-ChIP-seq | GSE79033 | Fang et al. 2016 |
| H3K27ac-ChIP-seq | GSE79033 | Fang et al. 2016 |
| H3K4ac-ChIP-seq | GSE79033 | Fang et al. 2016 |
| H3K9me1-ChIP-seq | GSE79033 | Fang et al. 2016 |
| H3K27me3-ChIP-seq | GSE79033 | Fang et al. 2016 |
| H3K9me3-ChIP-seq | GSE79033 | Fang et al. 2016 |
| H4K16ac-ChIP-seq | GSE69426 | Lu et al. 2015 |
| H3K23ac-ChIP-seq | GSE69426 | Lu et al. 2015 |
| H3K9me2-ChIP-seq | GSE81436 | Tan et al. 2016 |

## Slide 10
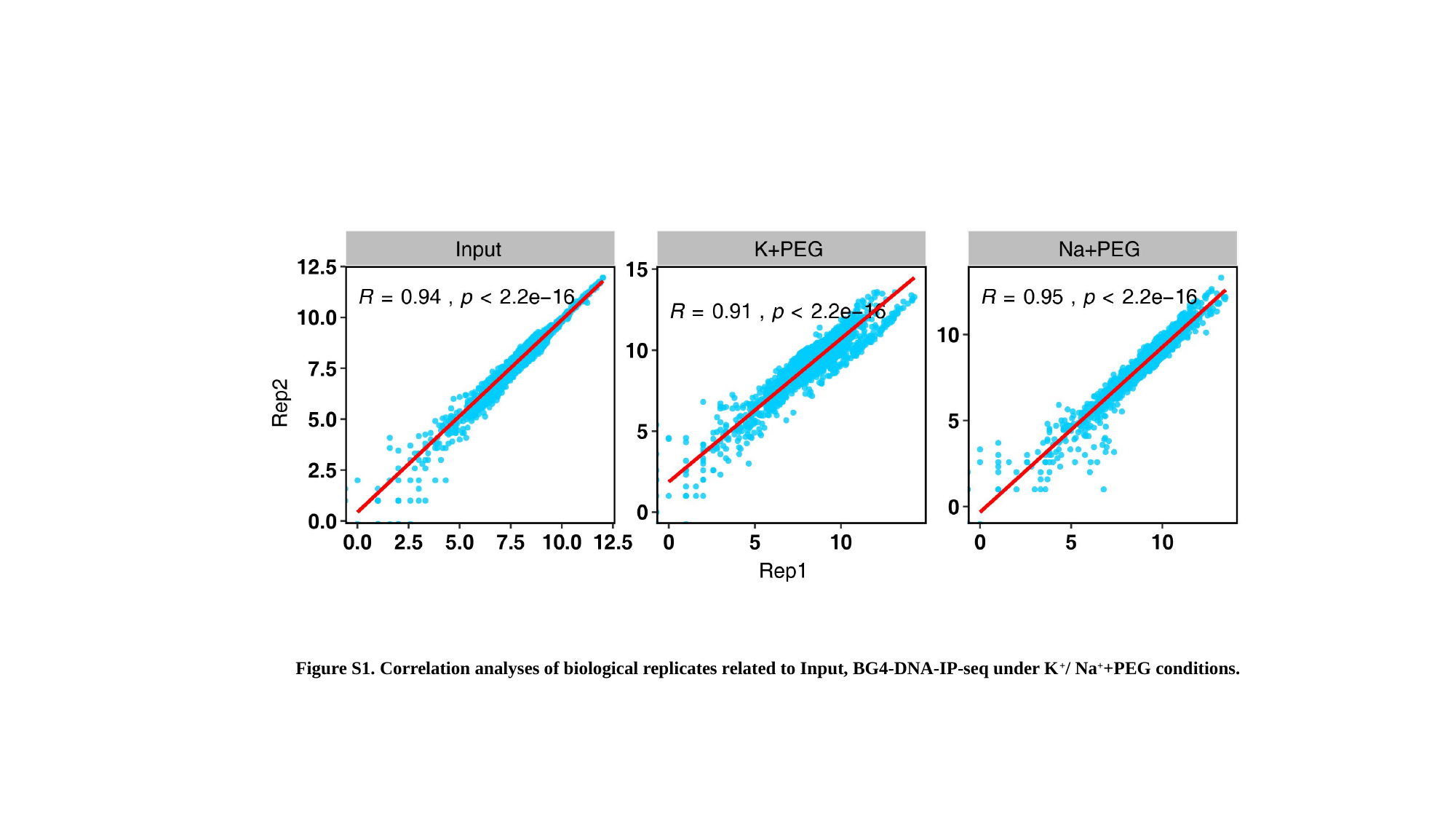

Figure S1. Correlation analyses of biological replicates related to Input, BG4-DNA-IP-seq under K+/ Na++PEG conditions.

## Slide 11
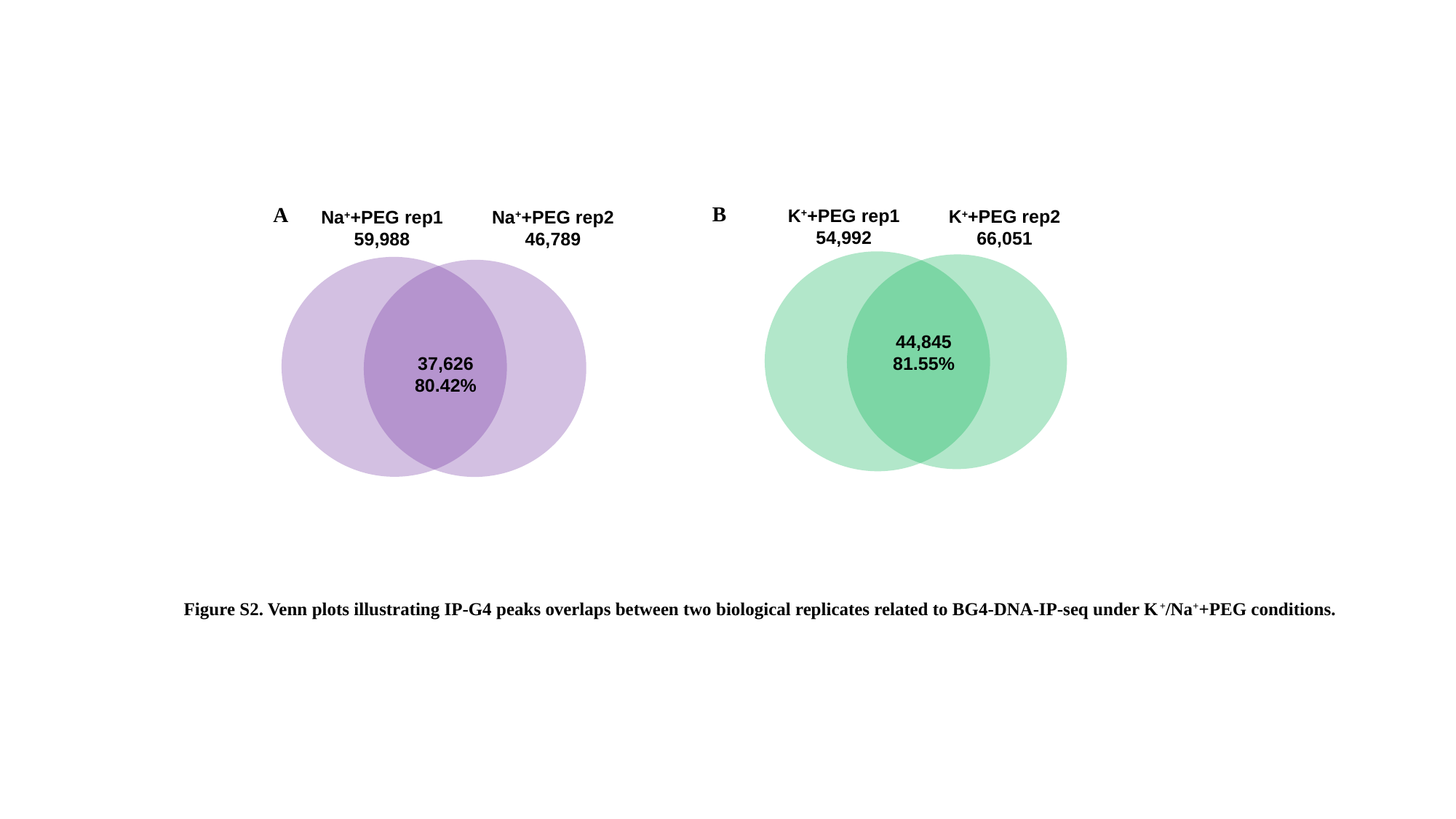

B
A
K++PEG rep1
54,992
K++PEG rep2
66,051
44,845
81.55%
Na++PEG rep2
46,789
Na++PEG rep1
59,988
37,626
80.42%
Figure S2. Venn plots illustrating IP-G4 peaks overlaps between two biological replicates related to BG4-DNA-IP-seq under K+/Na++PEG conditions.

## Slide 12
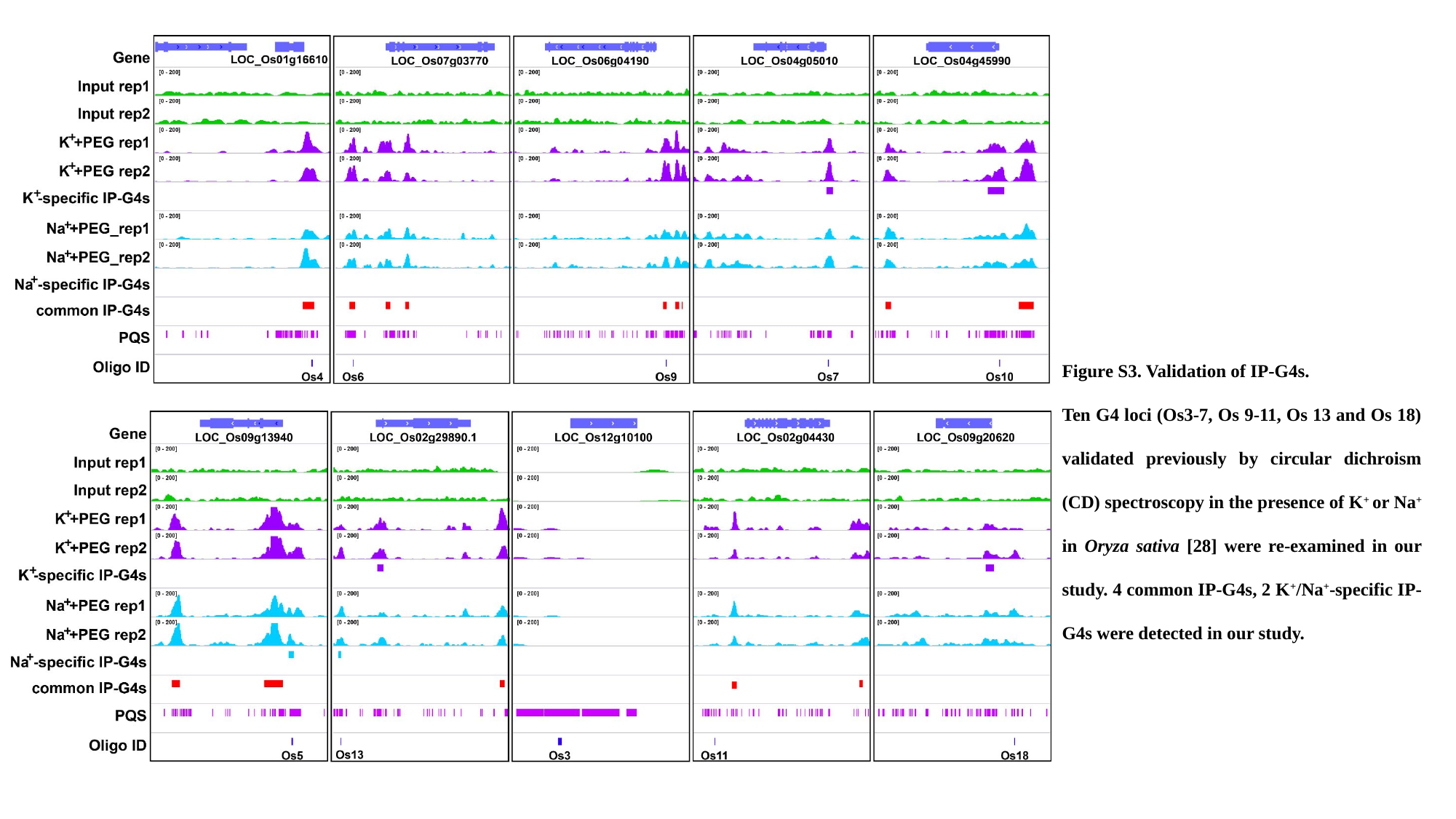

Figure S3. Validation of IP-G4s.
Ten G4 loci (Os3-7, Os 9-11, Os 13 and Os 18) validated previously by circular dichroism (CD) spectroscopy in the presence of K+ or Na+ in Oryza sativa [28] were re-examined in our study. 4 common IP-G4s, 2 K+/Na+-specific IP-G4s were detected in our study.

## Slide 13
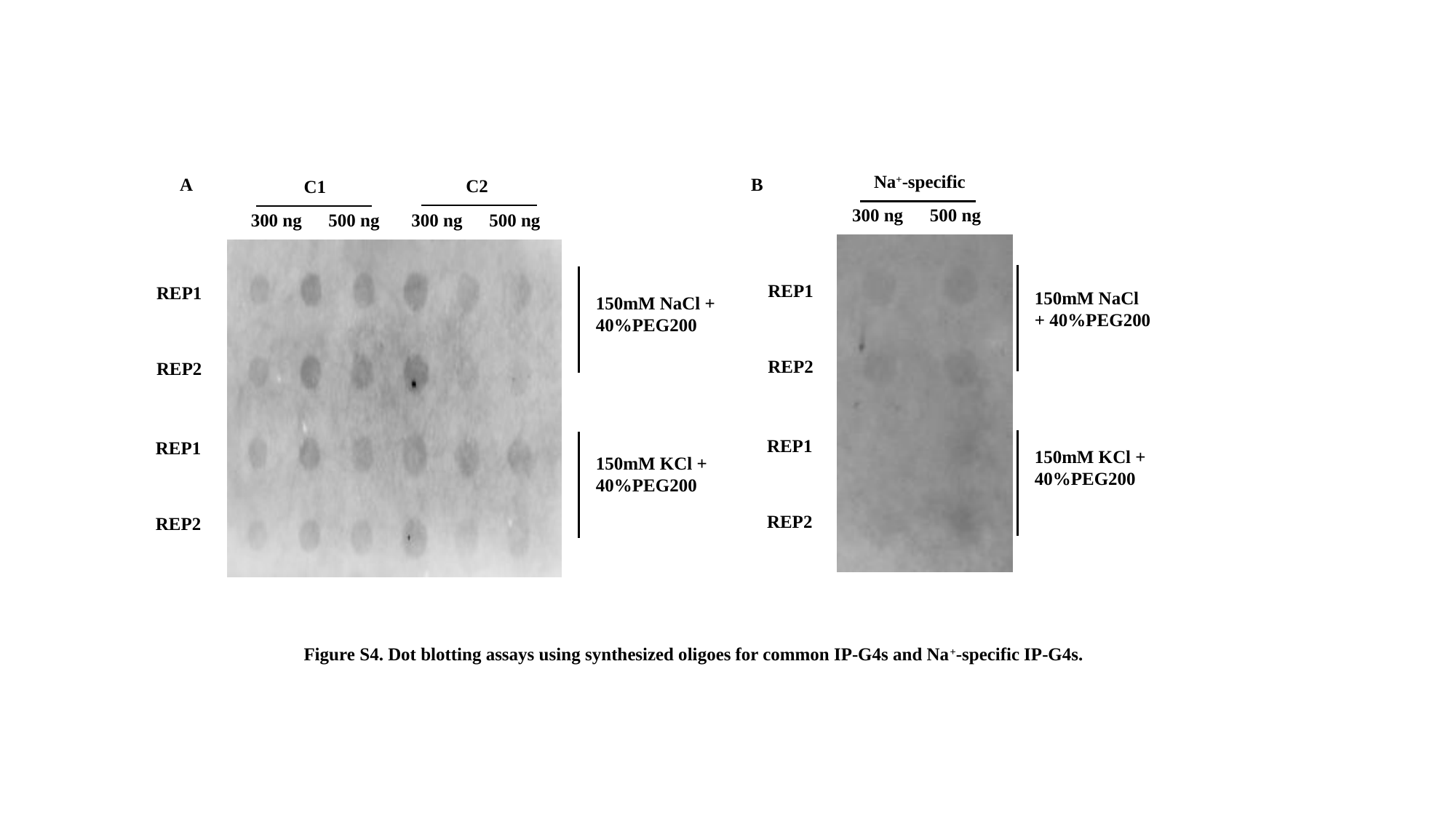

Na+-specific
300 ng
500 ng
REP1
REP2
REP1
REP2
150mM NaCl + 40%PEG200
150mM KCl + 40%PEG200
A
B
C2
C1
300 ng
500 ng
300 ng
500 ng
REP1
REP2
REP1
REP2
150mM NaCl + 40%PEG200
150mM KCl + 40%PEG200
Figure S4. Dot blotting assays using synthesized oligoes for common IP-G4s and Na+-specific IP-G4s.

## Slide 14
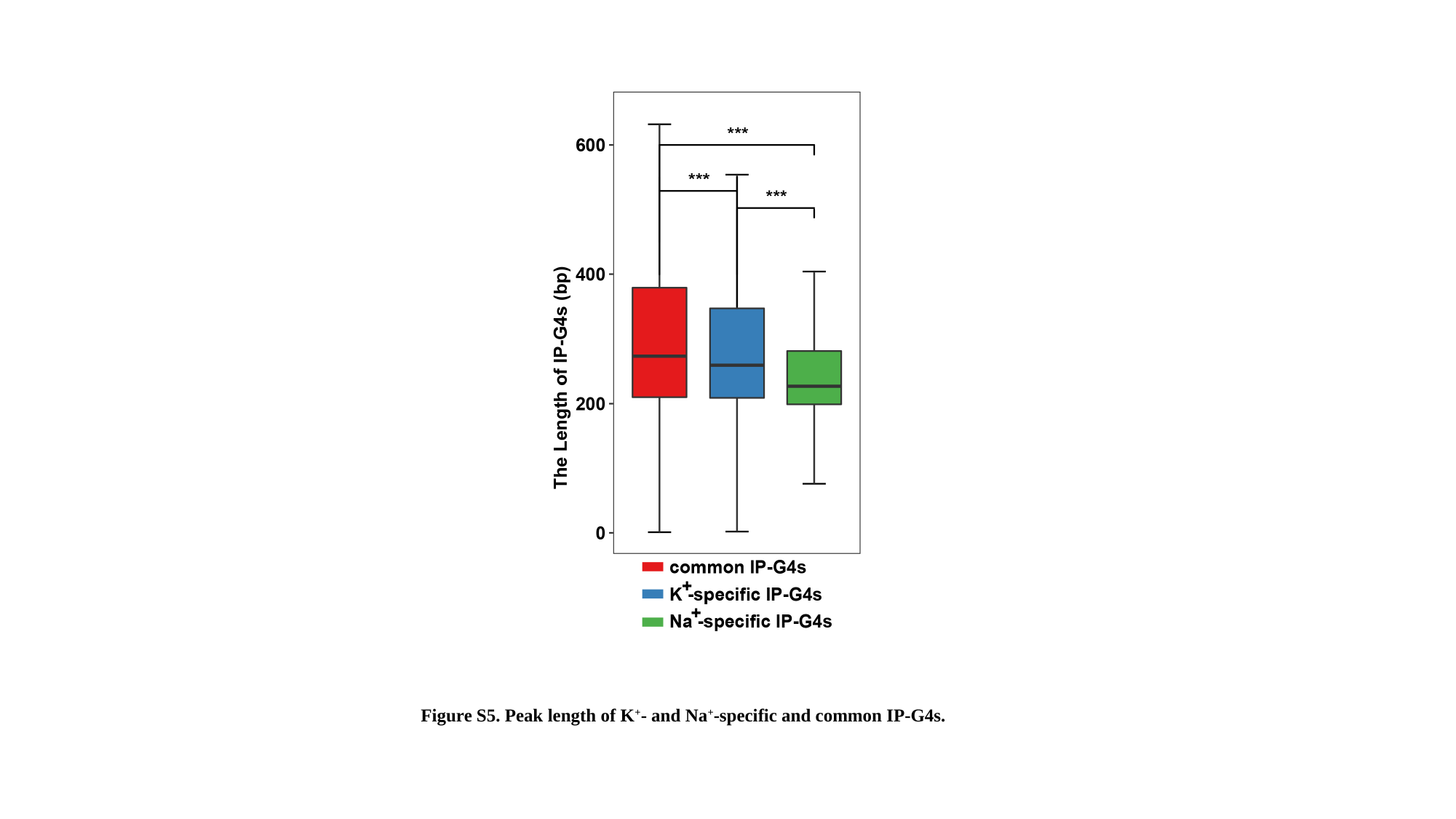

Figure S5. Peak length of K+- and Na+-specific and common IP-G4s.

## Slide 15
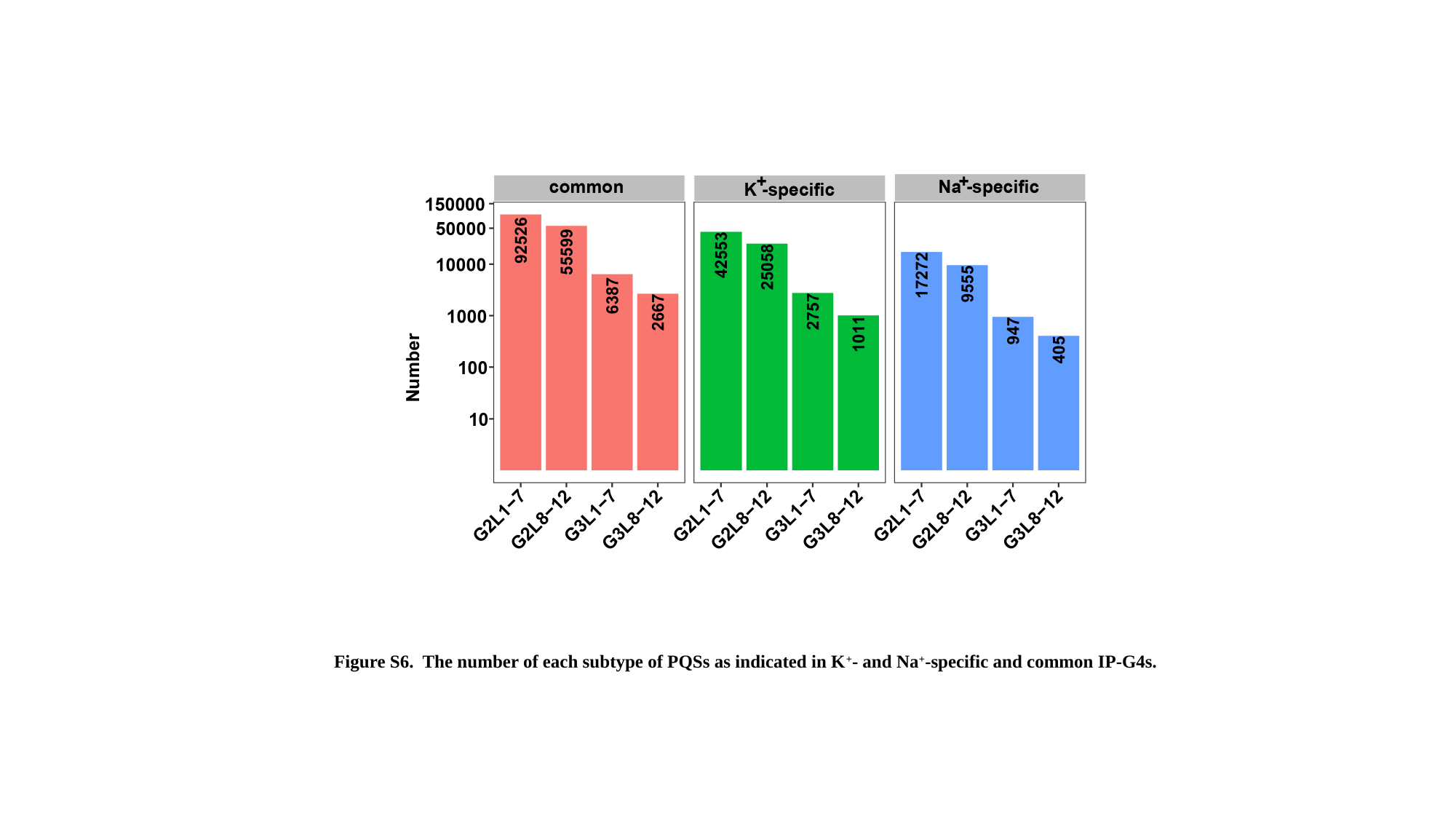

Figure S6. The number of each subtype of PQSs as indicated in K+- and Na+-specific and common IP-G4s.

## Slide 16
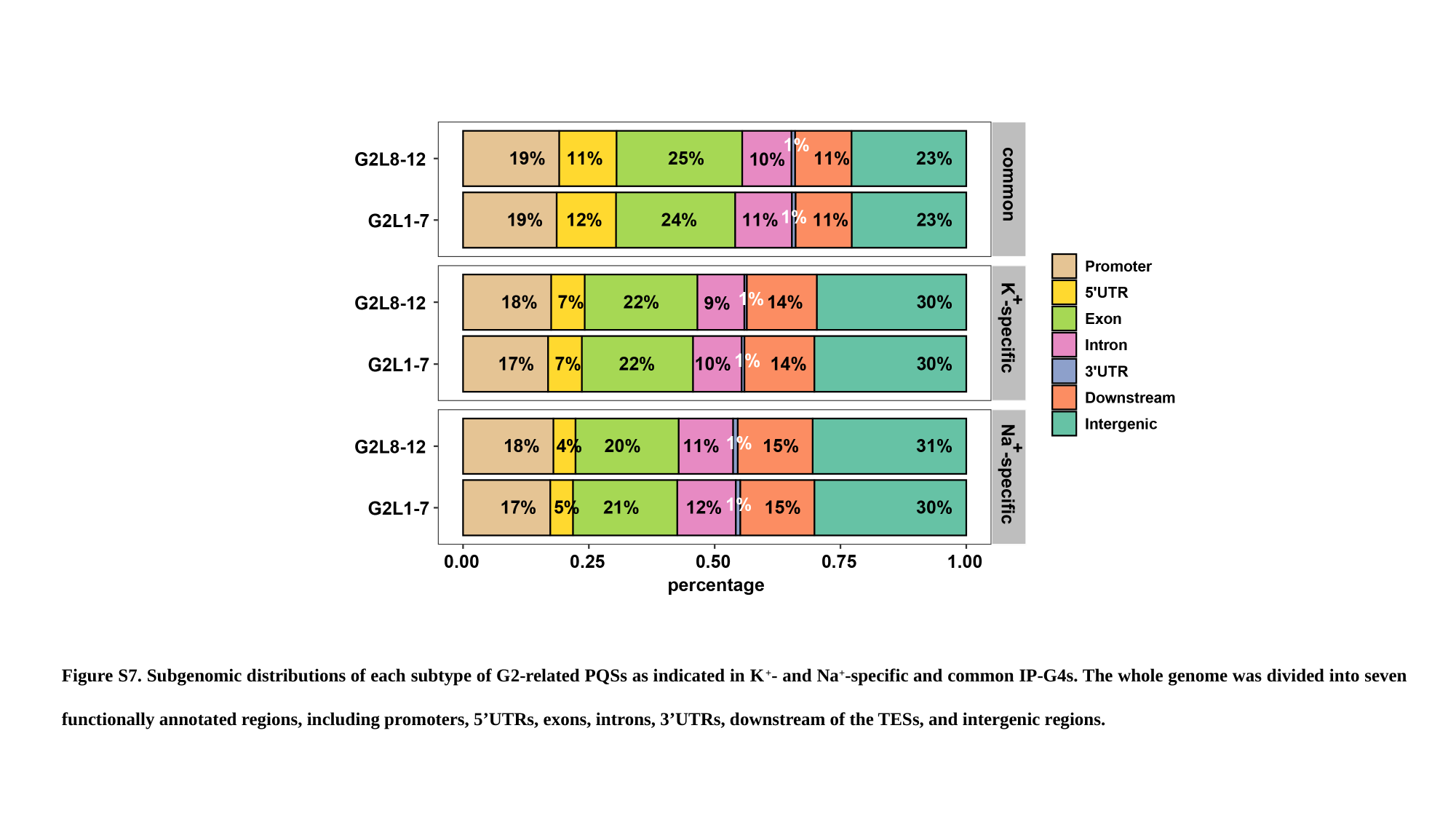

Figure S7. Subgenomic distributions of each subtype of G2-related PQSs as indicated in K+- and Na+-specific and common IP-G4s. The whole genome was divided into seven functionally annotated regions, including promoters, 5’UTRs, exons, introns, 3’UTRs, downstream of the TESs, and intergenic regions.
